# Randomised controlled cognition trials in remitted patients with mood disorders published between 2015 and 2021: A systematic review by the International Society for Bipolar Disorders Targeting Cognition Task Force

**DOI:** 10.1111/bdi.13193

**Published:** 2022-02-24

**Authors:** Kamilla W. Miskowiak, Ida Seeberg, Mette B. Jensen, Vicent Balanzá‐Martínez, Caterina del Mar Bonnin, Christopher R. Bowie, Andre F. Carvalho, Annemieke Dols, Katie Douglas, Peter Gallagher, Gregor Hasler, Beny Lafer, Kathryn E. Lewandowski, Carlos López‐Jaramillo, Anabel Martinez‐Aran, Roger S. McIntyre, Richard J. Porter, Scot E. Purdon, Ayal Schaffer, Paul Stokes, Tomiki Sumiyoshi, Ivan J. Torres, Tamsyn E. Van Rheenen, Lakshmi N. Yatham, Allan H. Young, Lars V. Kessing, Katherine E. Burdick, Eduard Vieta

**Affiliations:** ^1^ Copenhagen Affective Disorder Research Centre (CADIC) Psychiatric Centre Copenhagen Copenhagen University Hospital Copenhagen Denmark; ^2^ Department of Psychology University of Copenhagen Copenhagen Denmark; ^3^ Teaching Unit of Psychiatry and Psychological Medicine Department of Medicine University of Valencia CIBERSAM Valencia Spain; ^4^ Clinical Institute of Neuroscience Hospital Clinic University of Barcelona IDIBAPS CIBERSAM Barcelona Spain; ^5^ Department of Psychology Queen’s University Kingston Canada; ^6^ IMPACT Strategic Research Centre (Innovation in Mental and Physical Health and Clinical Treatment) Deakin University Geelong Vic. Australia; ^7^ Department of Old Age Psychiatry GGZ in Geest Amsterdam UMC, Location VUmc Amsterdam Neuroscience Amsterdam Public Health Research Institute Amsterdam The Netherlands; ^8^ Department of Psychological Medicine University of Otago Christchurch New Zealand; ^9^ Translational and Clinical Research Institute Faculty of Medical Sciences Newcastle University Newcastle‐upon‐Tyne UK; ^10^ Psychiatry Research Unit University of Fribourg Fribourg Switzerland; ^11^ Bipolar Disorder Research Program Institute of Psychiatry Hospital das Clinicas Faculdade de Medicina Universidade de São Paulo São Paulo SP Brazil; ^12^ McLean Hospital Schizophrenia and Bipolar Disorder Program Belmont Massachusetts USA; ^13^ 1811 Department of Psychiatry Harvard Medical School Boston Massachusetts USA; ^14^ 27983 Research Group in Psychiatry Department of Psychiatry Universidad de Antioquia Medellín Colombia; ^15^ Mood Disorders Psychopharmacology Unit, Brain and Cognition Discovery Foundation University of Toronto Toronto Canada; ^16^ Department of Psychiatry University of Alberta Edmonton Canada; ^17^ Department of Psychiatry University of Toronto; ^18^ Department of Psychological Medicine Institute of Psychiatry, Psychology and Neuroscience King’s College London London UK; ^19^ Department of Preventive Intervention for Psychiatric Disorders National Institute of Mental Health National Center of Neurology and Psychiatry Tokyo Japan; ^20^ Department of Psychiatry University of British Columbia Vancouver Canada; ^21^ Melbourne Neuropsychiatry Centre Department of Psychiatry University of Melbourne Carlton Australia; ^22^ Centre for Mental Health Faculty of Health, Arts and Design Swinburne University Australia; ^23^ Department of Clinical Medicine University of Copenhagen Copenhagen Denmark; ^24^ Department of Psychiatry Brigham and Women’s Hospital Boston Massachusetts USA

**Keywords:** bipolar disorder, cognitive impairment, intervention, ISBD Task Force, major depressive disorder, randomised controlled trials, recommendations, systematic review

## Abstract

**Background:**

Cognitive impairments are an emerging treatment target in mood disorders, but currently there are no evidence‐based pro‐cognitive treatments indicated for patients in remission. With this systematic review of randomised controlled trials (RCTs), the International Society for Bipolar Disorders (ISBD) Targeting Cognition Task force provides an update of the most promising treatments and methodological recommendations.

**Methods:**

The review included RCTs of candidate pro‐cognitive interventions in fully or partially remitted patients with major depressive disorder or bipolar disorder. We followed the procedures of the Preferred Reporting Items for Systematic reviews and Meta‐Analysis (PRISMA) 2020 statement. Searches were conducted on PubMed/MEDLINE, PsycInfo, EMBASE and Cochrane Library from January 2015, when two prior systematic reviews were conducted, until February 2021. Two independent authors reviewed the studies with the Revised Cochrane Collaboration's Risk of Bias tool for Randomised trials.

**Results:**

We identified 16 RCTs (*N* = 859) investigating cognitive remediation (CR; *k* = 6; *N* = 311), direct current or repetitive magnetic stimulation (*k* = 3; *N* = 127), or pharmacological interventions (*k* = 7; *N* = 421). CR showed most consistent cognitive benefits, with two trials showing improvements on primary outcomes. Neuromodulatory interventions revealed no clear efficacy. Among pharmacological interventions, modafinil and lurasidone showed early positive results. Sources of bias included small samples, lack of pre‐screening for objective cognitive impairment, no primary outcome and no information on allocation sequence masking.

**Conclusions:**

Evidence for pro‐cognitive treatments in mood disorders is emerging. Recommendations are to increase sample sizes, pre‐screen for impairment in targeted domain(s), select one primary outcome, aid transfer to real‐world functioning, investigate multimodal interventions and include neuroimaging.

## 1 INTRODUCTION

Cognitive impairments in attention, memory and executive functions occur across several neuropsychiatric disorders, including bipolar disorder (BD) and major depressive disorder (MDD). The profile of the cognitive impairment is similar across these disorders, involving non‐specific deficits in several domains,^1^, ^2^, ^3^ although the severity of impairment is greater in BD than in MDD.^4^ Cognitive impairments are not reversed by antipsychotic, antidepressant or mood‐stabilising treatments but persist during clinical remission in a substantial subset of patients^5^, ^6^, ^7^, ^8^ and are further compounded by alcohol/drug misuse and medical comorbidites.^9^, ^10^, ^11^ This contributes to socio‐occupational disability,^12^, ^13^, ^14^, ^15^ the largest socio‐economic burden of these disorders.^16^, ^17^ Cognitive impairment is also associated with poorer overall treatment response in mood disorders^18^, ^19^ including increased risk of manic relapse in BD.^20^, ^21^, ^22^ Given this, targeting cognitive impairment is a pressing treatment priority in mood disorders.^23^, ^24^ Accordingly, the field has undertaken a number of treatment trials over the last two decades, which aimed to improve cognition in these patients. Notwithstanding these efforts, there are still no clinically available pro‐cognitive treatments with replicated efficacy in remitted patients with BD or MDD.^25^, ^26^, ^27^, ^28^

Two systematic reviews from 2015 of randomised controlled trials (RCTs) in BD and MDD, respectively, found promising preliminary evidence for a series of behavioural, pharmacological and other biological interventions.^26^, ^27^ In BD, cognitive remediation (CR) and pharmacological interventions with either mifepristone, galantamine, insulin, erythropoietin (EPO), *Withania somnifera* or citicoline improved either a single or a subset of cognition measures, with effects of CR, mifepristone and EPO prevailing after Bonferroni correction.^26^ In MDD, preliminary effects were also observed in response to CR and EPO and additionally in trials of vortioxetine and transcranial direct current stimulation (tDCS).^27^ The risk of bias for the 41 RCTs included in these two reviews^26^, ^27^ was rated as high for 18 (44%), as moderate or unclear for 18 (44%) and low for only five (12%) of studies. Further, pseudospecificity (i.e., non‐specific cognitive improvement due to treatment‐associated decrease in mood symptoms) could not be ruled out because a substantial proportion of the identified RCTs were conducted in symptomatic patients (86% of trials in MDD and 37% in BD). A subsequent systematic review of RCTs in BD also concluded that the evidence for pro‐cognitive effects was mixed and uncertain given a scarcity of studies, small samples and high or unclear risk of bias in most trials.^29^ More recently, a meta‐analysis identified seven RCTs of CR or functional remediation.^30^ While cognitive gains were reported by most studies, the pattern of the improvements was heterogenous and not replicated across trials. All RCTs were evaluated as having moderate or high risk of bias. Taken together, the evidence for efficacy on cognition of behavioural, pharmacological and other biological interventions in mood disorders is mixed. Importantly, the reviews identified a series of common methodological issues that may have attenuated assay sensitivity in the trials.^26^, ^27^, ^30^

In prior work by the International Society for Bipolar Disorders (ISBD) Targeting Cognition Task Force, we examined the possible barriers to successful cognition trial outcomes and outlined methodological areas where a consensus was not yet established, including the need for pre‐screening for cognitive impairments, how to define efficacy outcomes, how to measure functional implications and how to manage mood symptoms and concomitant medications.^25^ Key recommendations from this work encouraged future studies to: (i) enrich samples for objectively measured cognitive impairments on neuropsychological tests, (ii) select global cognition as the primary outcome in general except for cases where there is evidence that a treatment is likely to target a specific cognitive domain, (iii) include a functional measure as co‐primary or key secondary outcome and (iv) enrol fully or partially remitted patients to avoid potential pseudospecificity issues due to concomitant mood improvements in response to the interventions, and (v) exclude patients with current substance or alcohol use disorders, neurological disease or unstable medical illness. Additionally, the Task Force suggested (vi) the implementation of neuroimaging assessments when possible and the systematic application of multimodal treatment approaches.^25^

The present systematic review by the ISBD Targeting Cognition Task Force is an update of the two previous systematic reviews of RCTs conducted in 2015 for BD and MDD, respectively.^26^, ^27^ Here, we focus on evidence from studies in *fully or partially remitted* patients to avoid pseudospecificity issues and examine efficacy on the trait‐related cognitive impairments, in line with the Task Force recommendations.^25^ The rationale for including RCTs published after January 2015 was to avoid overlap with the previous systematic reviews and to examine the most recent evidence and quality of the recent trials with the aim to update the Task Force recommendations. Specifically, with the current review, we aim to: (i) provide an update and critically evaluate the quality of the evidence from RCTs of candidate pro‐cognitive treatments *across mood disorders* in patients who are in full or partial remission published between January 2015 and February 2021, (ii) provide updated methodological recommendations, and (iii) outline the most promising targets for pro‐cognitive interventions. We did not conduct a quantitative meta‐analysis of the available evidence because of the discrepancies between types of interventions (including distinct psychological and pharmaceutical treatments), study designs (e.g., single‐ vs. double‐blind) and treatment schedules (single dose vs. months of treatment). Instead, this systematic review focuses on an evaluation of research design, methods and outcome criteria in the identified RCTs based on the Revised Cochrane Collaboration's Risk of Bias tool for Randomised trials (RoB2) and provides a discussion of the most promising targets for future research into pro‐cognitive interventions in mood disorders.

## 2 EXPERIMENTAL PROCEDURES

### 2.1 Data sources

This systematic review followed the procedures of the Preferred Reporting Items for Systematic Reviews and Meta‐analysis (PRISMA) 2020 statement.^31^ A comprehensive systematic computerised search was performed on the PubMed/MEDLINE, PsycInfo, EMBASE and Cochrane Library databases from 1st January 2015 to 28th February 2021. The search profile included four elements “Mood disorder”, “Cognition”, “Intervention” and “RCT” with each of their combinations and alternative keywords in the respective databases (see Supplementary material for details on the search profile). A protocol of the review was registered a priori in the online database, PROSPERO (registration number: CRD‐42021222836).

The initial search criteria were defined in accordance with the PICO framework (Population, Intervention, Comparison, Outcome). The clinical question was: In fully or partially remitted MDD or BD patients (population), are there any pharmacological or psychological pro‐cognitive interventions (interventions) that, when compared with either a passive control group (a waitlist condition with treatment as usual; TAU) or an active control group receiving another pro‐cognitive intervention (comparison), can improve cognitive functions (primary or secondary outcome)?

We included only original peer reviewed RCTs that aimed to improve objectively measured cognition through psychological, behavioural, pharmacological or other biological interventions in patients with MDD or BD in full or partial remission. Eligible reports involved (a) adult individuals (age ≥ 18) meeting either ICD or DSM diagnostic criteria for MDD or BD I or II (confirmed through a validated structured diagnostic interview) who were in *full or partial remission* at the time of baseline testing, as reflected by either Hamilton Depression Rating Scale 17‐items (HDRS‐17) score ≤16 or Montgomery–Asberg Depression Rating Scale (MADRS) <10 and – for BD samples – Young Mania Rating Scale (YMRS) score ≤14; (b) RCTs that investigated changes in cognition pre‐ and post‐intervention, with cognition as either a primary or secondary outcome; (c) RCTs reporting on primary prospective trial outcomes (i.e. not post‐hoc analyses of already published articles); (d) peer‐reviewed studies defined both at the journal websites and noted in the article with information on when it was received, revised and accepted; (e) articles published in English only. We excluded articles that: (i) examined samples with several diagnoses unless data for MDD or BD were reported separately, (ii) were non‐randomised trials or otherwise experimental trials, or (iii) were meeting abstracts, meta‐analyses, reviews and case reports. No specific criteria were applied to the format of the control arms because the RCTs involved diverse psychological and biological interventions and with different matched control conditions (e.g. placebo, TAU etc.).

### 2.2 Study selection

Two authors (IS and MBJ) independently performed a primary title/abstract screening for potentially eligible articles and, following this, a secondary full‐text screening was conducted. A hand‐search was performed as well by tracking and screening citations in the included articles for eligible articles. In all phases, all articles were considered in accordance with inclusion/exclusion criteria. No automation tools were used in the process. Interrater reliability was measured as percentage agreement, calculated as the number of *agreements* divided by the *total number of screened articles*. Agreement between the two authors was high (primary screening: 92%; secondary screening: 93%). Disagreements were discussed, and a consensus was reached in all cases through discussions with another author (KWM). Two authors (IS and KWM) extracted the measures of interest and summarised these in Tables 1 and 2. The data items were predefined according to the aims of the review and included the following: Authors, year of publication, study design, comparison, group, age, gender, mood state at entry, neurocognitive outcome measures and main findings. The syntheses of the included studies were predefined according to type of intervention, i.e. studies investigating the effect of cognitive remediation treatments (Table 1) and studies investigating the effect of pharmacological or brain stimulation treatments (Table 2).

**TABLE 1:** Studies investigating the effect of randomised controlled trials of cognitive remediation treatments across patients with mood disorders in full or partial remission published between January 2015 and February 2021

Author	Study design	Comparison (intervention/Control)	Group	Age (mean ± SD/median [IQR])	Gender (% F)	Mood state at entry (scale, mean ± SD/median [IQR])	Neurocognitive outcome measures	Main findings
Lewandowski et al. (2017)	RCT (Double‐blind)	70‐hours of Computerised Cognitive Remediation	39 BD	29.3 ± 7.5	51%	MADRS 1.8 ± 7.5 YMRS 5.6 ± 4.9	Primary outcome: The MCCB composite Additional outcomes: The 10 individual MCCB tests measuring processing speed, attention, working memory, verbal learning, visual learning, problem solving and social cognition	Linear mixed effects models revealed significant group‐by‐time interactions at post‐treatment for the cognitive composite and visual learning and memory and a trend for processing speed indicating significant improvements of cognitive remediation therapy over control
		70‐hours of Computer Control	33 BD	29.8 ± 9.2	58%	MADRS 2.2 ± 7.2 YMRS 4.7 ± 4.5		
Semkovska et al. (2017)	RCT	5 weeks (20‐hours) of Computerised Cognitive Remediation	11 MDD	45.9 ± 6.7	82%	HDRS 4.5 ± 2.3	Primary outcome: Not specified Additional outcomes: 11 tests that tapped into: psychomotor speed (DSST); divided attention (the d2 Selective attention test); auditory attention (the Digit Span Forward); verbal working memory (the Digit Span Backward); verbal learning and retention (the Logical memory‐I&II); visual learning, immediate recall and retention. The Delis–Kaplan Executive Function System's subtests were also used, including the assessment of the following executive functions: verbal fluency (three consecutive categories) for self‐regulation under external constraints, fluency switching for mental flexibility, towers for planning	Between‐group ANOVA revealed significant group‐by‐time interactions at post treatment in divided attention, verbal working memory, and planning, as well as on non‐targeted domains including long‐term verbal memory and switching organisation of own thinking abilities (five of 11 measures)
		5 weeks (20‐hours) of Computer Control	11 MDD	46.9 ± 9.3	82%	HDRS 4.0 ± 2.8		
Gomes et al (2019)	RCT (Single‐blind)	12 sessions of group‐based Cognitive Remediation	20 BD	42.7 ± 10.2	80%	N.I.	Cognition was secondary outcomes*: 10 neurocognitive tests from the CANTAB including 27 measures: MOT, RVP, RTI, SSP, SWM, OTS, PRM, DMS, AST, ERT *The primary outcome was time to first full mood episode	The effect on the primary outcome was not reported CR had no effect on quality of life or functioning Independent‐sample Student's t‐tests or Mann–Whitney tests of differential change between groups over time showed CR‐related improvement in five cognition measures, tapping into response times and visual memory and facial expression recognition
			19 BD	42.5 ± 10.2	58%	N.I.		
Strawbridge et al (2020)	RCT (Single‐blind)	12 weeks of Metacognition‐informed, therapy‐led, computerised cognitive remediation therapy	29 BD	43 [19]	72%	HDRS 4 [4] YMRS 2 [3]	Primary outcome: Psychomotor speed (DSST) Additional outcomes: Processing speed and attention (The Digit symbol substitution test and the Symbol search test); working memory (digit span); verbal learning (Verbal paired associates I); memory (verbal paired associates II); current IQ (WAIS); verbal fluency (FAS); executive function (Hotel test)	Linear mixed effects models revealed no significant group‐by‐time interaction in the primary cognition outcome (DSST) or in global cognition from pre‐ to post‐treatment. However, CRT‐related improvements were seen on tests of working memory, IQ and executive function (three of nine cognition measures)
		TAU	31 BD	42.5 [20]	65%	HDRS 3 [4.5] YMRS 1 [3.0]		
Listunova et al. (2020)	RCT (Single‐ blind)	5 weeks (3 × weekly) of Individualised Cognitive remediation	20 MDD	45.9 ± 11.3	75%	HDRS 9.2 ± 4.1	Primary outcome: A global cognitive composite score Additional outcomes: Tests tapping into information processing speed (The Trail Making A and Zahlen Digit Symbol Coding), attention (Alertness, Divided Attention and Selective Attention from the Vienna Test System), learning and memory (CVLT and Figural Memory Test), executive functions (Inhibition, The Trail Making‐B, Tower of London‐F, N‐back verbal from the Vienna Test System)	No effect was observed on the primary composite cognition measure However, general linear models revealed significant group‐by‐time interactions at post‐treatment for attention, showing that the two active groups improved relative to the TAU group
		5 weeks (3 × weekly) of Generalised Cognitive remediation	18 MDD	45.3 ± 15.1	78%	HDRS 8.7 ± 4.8		
		TAU	19 MDD	44.9 ± 10.3	68%	HDRS 11.8 ± 4.8		
Ott et al. (2020)	RCT (Single‐ blind)	10 weeks (2 × weekly) of Action‐Based Cognitive Remediation Therapy	32 BD	36[20]	72%	HDRS 6[4] YMRS 1.5[5]	Primary outcome: A global cognitive composite of: The Rey auditory verbal learning test (total recall); RBANS coding; Verbal fluency letter D; WAIS‐III letter‐number sequencing; Trail Making B; and the following Rapid Visual Processing (RVP) test from CANTAB Secondary cognition outcome: Executive function (One Touch Stocking of Cambridge) Tertiary outcomes: Individual measures comprising the primary outcome plus additional RAVLT measures, Verbal fluency letter S, Trail Making A, spatial working memory (CANTAB), RBANS digit span	No significant improvement was observed in the primary cognitive composite score. However linear mixed effects models revealed significant group‐by‐time interactions at post‐treatment on executive functioning (secondary outcome), which remained significant following adjustment for multiple comparisons across the secondary outcomes
		10 weeks (1 × weekly) of Control Treatment	29 BD	37[22]	76%	HDRS 6[4] YMRS 1[5]		

Abbreviations: N.I., No information; BD, Bipolar Disorder; MDD, Major Depressive Disorder; F, Female; HC, healthy controls; HDRS, Hamilton depression rating scale; YMRS, Young Mania rating scale; IQR, Inter quartile range; IU, International units; RCT, Randomised controlled trial; SD, standard deviation; TAU, Treatment as usual; MCCB, MATRICS Consensus Cognitive Battery; RBANS, Repeatable Battery for the Assessment of Neuropsychological Status; DSST, Digit Symbol Substitution Test;; CANTAB, Cambridge Neuropsychological Test Automated Battery; CVLT, California Verbal Learning Test; MOT; Motor Screening Task; RVP, Rapid Visual Information Processing; RTI, Reaction Time; SSP, Spatial Span; SWM, Spatial Working Memory; OTS, One Touch Stockings of Cambridge; PRM: Pattern Recognition Memory; DMS, Delayed Matching to Sample; AST, Attention Switching Task; ERT, Emotion Recognition Task.

**TABLE 2:** Studies investigating the effect of randomised controlled trials of pharmacological or brain stimulation treatments across patients with mood disorders in full or partial remission published between January 2015 and February 2021

Author	Study design	Comparison (intervention/control)	Group	Age (mean ± SD/median [IQR])	Gender (% F)	Mood state at entry (scale, mean ± SD/median [IQR])	Neurocognitive outcome measures	Main findings
Transcranial current and repetitive transcranial magnetic stimulation								
Bersani et al. (2017)	RCT (Double‐blind)	15 sessions over 3 weeks Prefronto‐Cerebellar Transcranial Direct Current stimulation	21 BD	48.1 ± 10.7	38%	HDRS 4.7 ± 1.8 YMRS 3.9 ± 1.2	Primary outcome: Not defined Additional cognition outcomes: Sustained attention (Trail Making Test‐A); executive functioning (Wisconsin Card Sorting Test, Trail Making Test‐B, Rey Complex Figure Test copy version); visuo‐spatial memory (Rey Complex Figure Test delay recall)	Between‐group ANOVA revealed significant group‐by‐time interactions for on executive function and visuospatial memory (two of five cognitive outcomes)
		Sham	21 BD	49.2 ± 10.2	71%	HDRS 4.7 ± 1.7 YMRS 4.4 ± 1.4		
Kumar et al. (2020)	RCT (Double‐blind)	10 sessions over 2 weeks, Bilateral Dorsolateral Prefrontal Cortex Anodal Transcranial Direct Current stimulation	18 MDD	66.3 ± 5.8	72%	MADRS 2.8 ± 2.53	Primary outcome: Global cognitive composite. Additional outcomes: Working memory (Computerised N‐back task), attention and psychomotor speed (Trail Making A, DSST), learning and memory (CVLT and Brief Visuospatial Memory Test) executive functions (Stroop Neuropsychological Screening, COWAT, Trail Making B, visuospatial skills (Clock Drawing Test)	No significant findings
		Sham	15 MDD	66.7 ± 5.8	60%	MADRS 4.3 ± 3.1		
Yang et al. (2019)	RCT (Single‐blind)	10 sessions over 2 weeks High‐frequency repetitive transcranial magnetic stimulation	25 BD	28.6 ± 8.1	52%	HDRS 4.8 ± 2.8 YMRS 0.8 ± 1.1	Primary outcome: Not specified Additional outcomes: 10 tests from the MCCB measuring processing speed, attention, working memory, verbal learning, visual learning, problem solving, and social cognition	Between‐group ANOVA revealed significant group‐by‐time interactions for working memory and speed of processing (two of 10 measures)
		Sham	27 BD	27.4 ± 7.1	30%	HDRS 4.9 ± 2.9 YMRS 1.0 ± 1.3		
Pharmacological Interventions								
Alda et al. (2017)	RCT crossover (Double‐blind)	12 weeks of Methylene blue (195 mg) versus Placebo (low dose methylene blue; 15 mg)	Crossover total: 37 BD	48.3 ± 9.2	74%	HDRS 7.8 ± 4.5 YMRS 2.9 ± 3.2	Cognition was secondary outcome*: A battery including memory (CVLT and a process‐dissociation task); executive function (Trail Making Test B); selective attention (NS); negative priming (NS); inhibition of return (NS) *The primary outcome was residual mood symptoms	No significant findings.
Ciappolino et al. (2020)	RCT (Double‐blind)	12 weeks of Docosahexaenoic acid supplementation (5 capsules with 1250 mg/day)	13 BD	36 ± 12	77%	HDRS <8 and YMRS <3 (otherwise not reported)	Primary outcome: Not specified Additional outcomes: The BAC‐A including eight tests: two investigating the emotional domain, which include, (i) Affective Processing Test, which evaluates components of immediate and delayed affective and non‐affective memory, and (ii) Emotion Inhibition Test, which measures the ability to suppress an automatic process, like reading, and the irrelevant elaboration of the word's meaning (affective processing) in a colour naming task, and six exploring the cognitive/linguistic domain, including, (iii) List Learning, a measure of verbal learning and memory, (iv) Digit Sequencing Task, which evaluates working memory, (v) Token Motor Task, which estimates visuo‐motor abilities, (vi) Verbal Fluency, used to evaluate both semantic and phonemic fluency, (vii) Symbol‐Coding Task, employed to measure attention and processing speed, (viii) Tower of London, which provides an estimation of problem‐solving abilities, a subcomponent of executive functions	No significant findings
		Placebo	18 BD	50.4 ± 11.3	67%			
Kaser et al. (2017)	RCT (Double‐blind)	Single‐dose of Modafinil (200 mg)	30 MDD	43.97 ± 11.03	63%	MADRS 4.6 ± 2.7	Primary outcome: Eight measures from the following four tests from the CANTAB: Paired Associates Learning, One Touch Stockings of Cambridge, Spatial Working Memory and Rapid Visual Information Processing Secondary outcomes: Nine CANTAB measures from the above four tests	Between‐group ANOVA revealed significant group‐by‐time interactions for measures of episodic memory and working memory
		Placebo	30 MDD	46.10 ± 10.69	60%	MADRS 4.5 ± 3.2		
Yatham et al. (2017)	RCT (Open‐label)	6 weeks of Lurasidone as adjunctive therapy (20–80 mg/day)	17 BD	38.7 ± 12.2	71%	MADRS 2.7 ± 2.8	Primary outcome: global cognition score based on the ISBD‐BANC consisting of: CVLT‐II trials 1–5, CVLT‐II delayed free recall, BVMT‐R trials 1–3, BVMT‐R delayed recall, TMT‐A time, TMT‐B time, Continuous Performance Test –Identical Pairs trials 1–3, Animal Naming Fluency, Letter‐Number Sequencing, Spatial Span, Symbol Coding, Stroop Word, Stroop Colour, and Stroop Colour‐Word	Between‐group ANOVA revealed significant group‐by‐time interactions at post‐treatment on the primary global cognition outcome
		TAU	17 BD	38.5 ± 10.1	65%	2.4 ± 2.9		
Van Meter	RCT (Double‐blind)	8 weeks of Pramipexole (initiated at 0.125 mg/day and increased to 4.5 mg/day)	31 BD	41.0 ± 14.3	58% (across entire cohort)	HDRS 4.8 ± 3.9 YMRS 2.7 ± 2.4	Primary outcome: The MCCB global composite Secondary outcome: Iowa Gambling Task Exploratory outcomes: MCCB domain scores	No significant findings
		8 weeks of Placebo	29 BD	37.8 ± 12.1		HDRS 6.4 ± 3.9 YMRS 2.6 ± 2.5		
Smith et al. (2018)	RCT (Double‐blind)	2 weeks of Vortioxetine (20 mg/day)	24 MDD	33.1 ± 9	67%	HDRS 1.0 ± 1.0	Cognition was secondary outcome*: A combined battery, including the following subtests: DSST, RAVLT, and Trail Making A and B *(the primary outcome was working memory‐related neural activity)	Group by time ANOVA revealed an improvement in the vortioxetine on one measure of attention (Trail Making A) but no other aspects of cognition
		2 weeks of Placebo	24 MDD	38.1 ± 8.8	46%	HDRS 1.6 ± 2.1		
Nierenberg et al. (2019)	RCT (Double‐blind)	8 weeks of Vortioxetine (10–20 mg/day) as add‐on to SSRI	52 MDD	45.9 ± 12.7	79%	HDRS 5.6 ± 2.3	Primary outcome: Psychomotor speed (DSST) Secondary outcomes: The RAVLT acquisition and delayed recall, Trail Making A and B; The Stroop Colour naming test congruent and incongruent; Simple Reaction Time; and Choice reaction time	No significant findings
		8 weeks of Vortioxetine (10–20 mg/day)	50 MDD	50.6 ± 10	69%	HDRS 6.1 ± 2.4		
		8 weeks of continued SSRI treatment	49 MDD	47.9 ± 11.5	68%	HDRS 5.6 ± 2.1		

Abbreviations: BAC‐A, Brief Assessment of Cognition in Affective Disorder; BD, Bipolar Disorder; UD, Unipolar Disorder; F, Female; HC, healthy controls; HDRS, Hamilton depression rating scale; YMRS, Young Mania rating scale; IQR, Inter quartile range; IU, International units; RCT, Randomised controlled trial; SD, standard deviation; TAU, Treatment as usual; MCCB: MATRICS Consensus Cognitive Battery; Brief Visuospatial Memory Test, BVMT; CANTAB, Cambridge Neuropsychological Test Automated Battery; CVLT, California Verbal Learning Test; RAVLT; Rey Auditory Verbal Learning Test; DSST, Digit Symbol Substitution Test; ISBD‐BANC, International Society for Bipolar Disorders Battery for Assessment of Neurocognition; COWAT, Controlled Oral Word Association.

### 2.3 Risk of bias assessment

The risk of bias within and across the included randomised controlled studies was assessed by two authors (IS and KWM) according to the Revised Cochrane Collaboration's Risk of Bias tool for Randomised trials (RoB2) (https://sites.google.com/site/riskofbiastool/welcome/rob‐2‐0‐tool/current‐version‐of‐rob‐2). The RoB2 assessment tool provided by Cochrane was used independently by the two authors. Table 2 displays the RoB2 evaluations of the included RCTs. To find any missing information in the included trials, additional searches for registered RCTs were performed on clinicaltrials.gov, and a search for published study protocols was also performed on relevant search engines. The PRISMA 2020 checklist was completed (supplementary material).

## 3 RESULTS

### 3.1 Study characteristics

**FIGURE 1:**
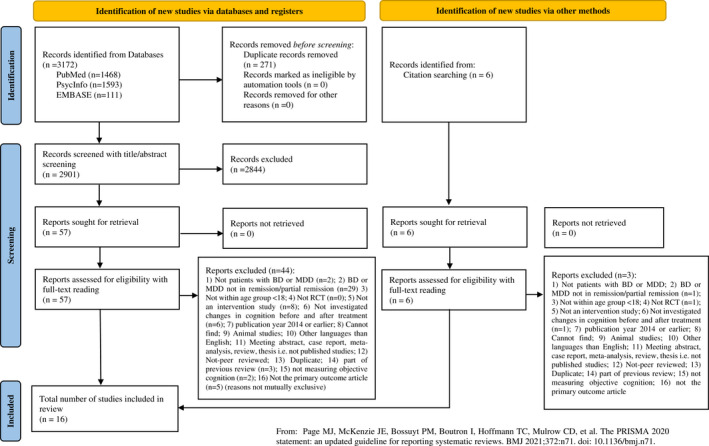
PRISMA 2020 flowchart

The systematic search, together with the additional hand‐search, identified 2907 articles (after removal of duplicates) that were included for title/abstract screening (primary screening). Of these, 63 were evaluated for eligibility via a full‐text reading (secondary screening). This resulted in the inclusion of 16 articles that met the inclusion criteria (see Figure 1). Tables 1 and 2 display the characteristics of the identified RCTs investigating potential pro‐cognitive psychological or biological treatments in patients with MDD or BD in full or partial remission (*N* = 859). Six studies investigated cognitive remediation (CR) interventions (*N* = 311),^32^, ^33^, ^34^, ^35^, ^36^, ^37^ three studies investigated transcranial current or repetitive magnetic stimulation (*N* = 127)^38^, ^39^, ^40^ and seven studies investigated pharmacological treatments (*N* = 421)^41^, ^42^, ^43^, ^44^, ^45^, ^46^, ^47^ of which three received support from the pharmaceutical industry.^44^, ^45^, ^47^ Five (31%) studies employed pre‐screening of objective cognitive impairments.^34^, ^35^, ^44^, ^46^, ^47^ Applied criteria in trials with global cognition as the primary outcome^34^, ^35^, ^46^, ^47^ were impairments on either: a global cognitive composite score,^34^, ^35^, ^46^ minimum two cognitive tests^34^, ^35^ or one of two tests.^47^ In a trial with a single cognitive domain (psychomotor speed) as the primary outcome, patients were enriched for deficits in that domain.^44^ Eight studies included additional assessments of psychosocial function,^32^, ^33^, ^34^, ^35^, ^37^, ^44^, ^47^, ^48^ three studies of functional capacity^33^, ^35^, ^37^ and six of subjective cognitive difficulties.^35^, ^37^, ^43^, ^44^, ^45^, ^47^

### 3.2 Candidate cognitive remediation interventions

Six studies examined the effect of CR as an add‐on to pharmacotherapy in partially remitted MDD or BD patients with samples ranging from *N* = 22–75 (Table 1). Of these, three studies evaluated computerised CR interventions.^33^, ^36^, ^37^ The first study investigated the effects of 70 hours of computerised CR (*n* = 39) relative to a computer control programmes (*n* = 33) three times weekly over 24 weeks in BD^33^ with global cognition as the primary outcome. The treatment adherence was high (96%). The active group showed significantly greater improvement with a large effect size than the control group in the primary global MCCB cognitive composite outcome in the absence of changes in subsyndromal mood symptoms. A treatment‐related improvement of a large effect size was also observed in the MCCB visual memory test, but not the six other tests. No corresponding improvement of psychosocial function or functional capacity was observed.

The second study assessed the impact of 20 hours of computerised CR over 5 weeks in MDD (*n* = 11) compared with a computer control programme (*n* = 11).^36^ It was not specified which cognition measure was the primary outcome, and psychosocial function was not investigated. Ninety‐five % of the participants completed the study. Computerised CR resulted in significantly greater improvement than the control treatment across divided attention and switching, verbal working memory, planning skills and verbal memory (on five of 11 measures; effect sizes not provided), in the absence of changes in subsyndromal mood symptoms. Of these, all but "divided attention" and "switching" prevailed after Bonferroni correction.

The third study examined the effects of 20–30 h of computerised metacognition‐informed, therapist‐led CR over 12 weeks in BD (*n* = 29) compared with TAU (*n* = 31) on cognitive functions, with psychomotor speed (Digit Symbol Substitution Test; DSST) specified as the primary outcome.^37^ The study completion rate was 88%. Patients in the CR group showed no greater improvement than the TAU group immediately after treatment in the primary cognition outcome or in a global cognition composite based on tests of verbal learning and memory, working memory and executive functions (for details, see Table 1). However, CR‐related improvements with moderate effect sizes were seen on tests of working memory, IQ and executive function (three of nine cognition measures), which all prevailed at a 3‐month follow‐up assessment. Subsyndromal symptoms were similar between groups, although the CR group had slightly higher subsyndromal depression symptoms at the 3‐month follow‐up. The observed cognitive improvements would, however, not have survived Bonferroni correction for multiple comparisons across the nine cognition measures. Notably, CR improved psychosocial functions and functional capacity, and the effects on psychosocial functions prevailed at a 3‐month follow‐up.

Three studies involved computerised training combined with group‐based CR sessions to aid the transfer of acquired skills to daily life cognitive challenges.^32^, ^34^, ^35^ The first study examined the effects of 15 sessions of CR conducted over 5 weeks in MDD with three weekly sessions in an individualised training format that targeted patients’ particular deficits (*n* = 20) or in a generalised training format (*n* = 18) in comparison with TAU (*n* = 19).^34^ Improvement of a global cognition composite was pre‐specified as the primary outcome, while attention, processing speed, learning and memory, and executive functioning were secondary outcomes. Ninety‐two percent of the participants completed the study. No treatment‐related improvement of the primary global cognition outcome was observed. Regarding the secondary cognition outcomes, the two CR groups showed greater improvement than the control group in attention with a large effect size, but not in any other cognition measures. CR‐treated patients also showed improved self‐reported psychosocial functioning in the absence of significant changes in subsyndromal mood symptoms.

The second study^32^ investigated the effects of 12 sessions of group‐based CR in BD (*n* = 20) vs. TAU (*n* = 19) with cognition being the secondary outcome (time to relapse was the primary outcome). CR improved response times, visual memory and some aspects of facial expression recognition, of which the effect on visual memory would have survived Bonferroni correction for multiple comparisons. No CR‐related improvement was observed in functioning or subjective cognition, whereas changes in subsyndromal mood symptoms were not reported.

The third study examined the effects of group‐based action‐based CR (ABCR) conducted in twice weekly sessions over 10 weeks (*n* = 32 BD) compared with 10 weekly unstructured control group meetings supervised by a therapist (*n* = 29 BD). A global cognition measure was defined as the primary outcome, executive function (One Touch Stocking of Cambridge; OTS) and psychosocial function as secondary outcomes and additional cognition measures, functional capacity and subjective cognitive difficulties as tertiary (exploratory) outcomes.^35^ Ninety‐five percent of the participants completed the study. No significant treatment‐related improvement was found on the global cognition outcome. However, the ABCR group displayed significantly greater improvement than the control group in the secondary executive function outcome with a large effect size, which prevailed after adjustment for multiple comparisons. Additional moderate‐to‐large ABCR‐related improvement was observed on verbal learning and memory, although this did not survive adjustment for multiple comparisons across all tertiary outcomes. Finally, ABCR‐treated patients reported improved subjective cognitive functioning in daily life – but showed no change in psychosocial function or functional capacity – compared with the control group. The effects occurred in the absence of treatment‐related changes in mood symptoms.

In summary, all six CR interventions showed promising results; one study was positive, as indicated by significant treatment effects on the primary (global) cognition outcome^33^; another study – with no pre‐specification of which cognition outcome was primary – showed improvements in 5 of 11 cognition measures, of which four would have survived Bonferroni correction.^36^ The final four studies^32^, ^34^, ^35^, ^37^ found no treatment benefits on the primary cognition outcomes but all revealed improvements in multiple secondary and tertiary cognition measures. Importantly, participants’ completion rates were high (88%–96%) in all studies, but one (65%)^32^ indicating good feasibility of CR in general. Three of the CR interventions – each of which involved explicit therapist techniques to facilitate transfer of cognitive skills to daily life – also improved either subjective cognitive functioning, psychological functioning or functional capacity.^34^, ^35^, ^37^

### 3.3 Other candidate biological interventions involving stimulation of the cortex

Three studies examined the cognitive benefits of transcranial direct current stimulation (tDCS) or repetitive transcranial magnetic stimulation (rTMS) as an add‐on to pharmacotherapy in partially remitted MDD or BD patients with sample sizes ranging from *n* = 33 to 52 (Table 2).^38^, ^39^, ^40^ None of the studies assessed psychosocial function or subjective cognition. One study investigated the effects of 15 20‐minutes sessions of prefronto‐cerebellar tDCS (*n* = 21) relative to sham (*n* = 21) delivered over 3 weeks to patients with BD.^38^ No primary cognition measure was defined a priori. All participants completed the study. The active tDCS group showed greater improvement of executive functioning and visuospatial memory than the sham group (i.e., two of five cognition measures; effect sizes not reported). However, the effects would not have survived Bonferroni adjustment for multiple comparisons. Changes over time in subsyndromal mood symptoms were not reported.

Another study investigated the effects of 10 sessions of bilateral dorsolateral prefrontal‐cortex anodal tDCS over 2 weeks (*n* = 18) relative to sham (*n* = 15) in older age MDD^39^ (Table 2). Global cognition was defined as the primary outcome and working memory as an exploratory outcome. In total, 97% of the participants completed the study. No significant effects of tDCS were found on the global cognitive composite based on a comprehensive neurocognitive test battery (see Table 2) or working memory, and no effects were observed on subsyndromal mood symptoms.

Finally, a study investigated the effects of 10 sessions high‐frequency rTMS over 2 weeks (*n* = 25) relative to sham (*n* = 27) in BD.^40^ It was not specified which cognition measure was the primary outcome. All the participants completed the study. The rTMS‐treated patients showed greater improvement than sham‐treated patients in working memory and speed of processing (two of ten cognition measures) with small effect sizes in the absence of changes in mood symptoms. However, these effects would not have survived adjustment for multiple comparisons.

Taken together, the studies showed no clear cognitive benefits; one tDCS study in MDD was negative, while the two other studies in BD (of tDCS and rTMS, respectively) showed selective treatment‐related cognitive improvement that would not have survived Bonferroni correction.

### 3.4 Candidate pharmacological interventions

Seven studies examined the potential cognitive benefits of add‐on pharmacological interventions in partially remitted MDD or BD patients with samples ranging *N* = 31–151 (Table 2).^41^, ^42^, ^43^, ^44^, ^45^, ^46^, ^47^ Three included assessments of psychosocial function^42^, ^44^, ^47^ and four of subjective cognition.^43^, ^44^, ^45^, ^47^ One crossover study in BD patients investigated the effects of methylene blue, an inhibitor of nitric oxide synthase with putative effects on neuroplasticity, which has also been found to improve hypotension. Methylene blue was administered in three doses per day for 12 weeks (*n* = 17) versus placebo (*n* = 20), with cognition measures as secondary outcomes (the primary outcome was residual mood symptoms).^41^ In total, 73% of the participants completed the study, with dropouts being primarily due to symptom fluctuations and mood episodes during the 6‐month long trial period. The study revealed no significant‐related cognitive improvements after methylene blue versus placebo treatment, despite beneficial effects on residual depression and anxiety symptoms (psychosocial function not assessed).

The second study investigated the effects of docosahexaenoic acid (DHA) supplementation, which is an omega‐3 fatty acid, with five capsules (1250 mg) DHA per day for 12 weeks (*n* = 13) versus placebo (*n* = 18) in BD (and in healthy controls).^42^ No cognition measure was defined as the primary outcome, and the attrition rate was also not specified. The study revealed no significant cognitive or functional improvements in DHA versus placebo‐treated patients and no associations between cognition and subsyndromal mood symptoms.

The third study investigated the acute effects of a single dose of modafinil (200 mg) (*n* = 30) versus placebo (*n* = 30) in MDD.^43^ Eight cognition measures from four computerised cognitive tests were defined as primary outcomes, while nine measures were defined as secondary outcomes. All participants completed the study. Modafinil‐treated patients showed enhanced episodic memory and working memory with medium to large effect sizes relative to those given placebo but not in other aspects of cognition. These effects occurred in the absence of group differences in subsyndromal mood symptoms or subjective cognitive change and prevailed after adjustment for multiple comparisons.

The fourth study investigated the effects of 6 weeks of lurasidone (*n* = 15) versus TAU (*n* = 15) in a randomised, open‐label, outcome‐assessor blind pilot study in BD, type I.^47^ The primary outcome was global cognition score based on the International Society for Bipolar Disorders Battery for Assessment of Neurocognition (ISBD‐BANC).^49^ The study found greater global cognitive improvements in the lurasidone‐treated patients than those in TAU with a large effect size. There were also significant improvements in subjective cognition but not psychosocial function in the lurasidone group compared with TAU. No concurrent change in subsyndromal mood symptoms was observed.

The fifth study investigated the effects of 8 weeks of pramipexole (initiated at 0.125 mg/day and increased to 4.5 mg/day; *n* = 31) versus placebo (*n* = 29) in fully remitted, objectively cognitively impaired patients with BD using a randomised, double‐blind design. No benefits of pramipexole were observed on the primary global cognition outcome, the MCCB,^46^ the secondary outcome, the Iowa Gambling Task or exploratory MCCB domain outcomes. No differences between groups were observed in mood changes over time (psychosocial function and subjective cognition were not assessed).

Finally, two studies investigated the effects of vortioxetine. One study investigated the effects of 2 weeks of vortioxetine (10–20 mg/day) (*n* = 24) versus placebo (*n* = 24) in MDD (and healthy controls) with cognition being a secondary outcome (the primary outcome was a neuroimaging‐based measure of neuronal activity during working memory performance).^45^ All participants completed the study. Vortioxetine improved one measure of attention in MDD patients (effect size not reported) but no other aspects of cognition, and this effect would have not survived correction for multiple comparisons. While no effects of vortioxetine were observed on clinician‐rated depression, vortioxetine‐treated patients displayed improvement in self‐rated depression relative to placebo‐treated patients. No effects of vortioxetine were seen on patients’ subjective cognition (psychosocial function not assessed). The other study investigated the effects of 8 weeks of vortioxetine (10–20 mg/day) as add‐on to selective serotonin reuptake inhibitors (SSRI) (*n* = 52) or as monotherapy (*n* = 50) versus continued SSRI monotherapy (*n* = 49) in MDD.^44^ Psychomotor speed (DSST) was the primary outcome, with additional measures of cognition, psychosocial function, functional capacity and subjective cognition being secondary outcomes. In total, 99% of the participants completed the study. The findings revealed no greater cognitive improvement with vortioxetine as add‐on to SSRI or as monotherapy compared with the SSRI monotherapy on the primary or secondary cognition outcomes – and no differential effects on subsyndromal depression symptoms, psychosocial function, functional capacity or subjective cognition.

Taken together, the pharmacological interventions for cognitive impairments showed limited evidence. Two studies of lurasidone or modafinil administration indicated cognitive benefits in BD and MDD, respectively, while the remaining five studies were negative.

### 3.5 Risk of bias evaluation

**FIGURE 2:**
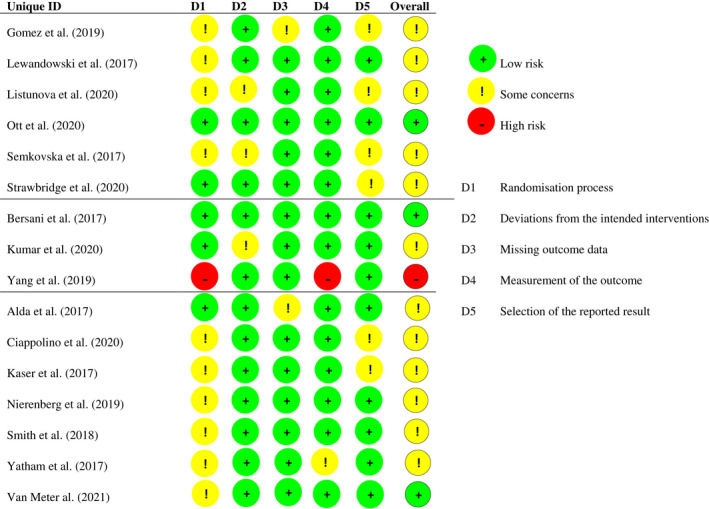
Risk of bias evaluations. Studies divided into cognitive remediation (first section), magnetic or direct current stimulation (second section) and pharmacological (third section) interventions and sorted alphabetically after the first author in each section

Figure 2 displays the risk of bias evaluations of the included RCTs. Twelve studies (75%) were evaluated as involving ‘some concerns’ (i.e., moderate/unclear risk of bias), one (6%) as having ‘high risk’ of bias and three (19%) as having ‘low risk of bias’. A common source of bias among the 13 studies for which cognition was the primary focus (rather than a secondary outcome), five (38%) had not selected *one primary cognition outcome* a priori, which introduced a risk of selective outcome reporting. Another common source of bias in 10 studies (62.5%) was a lack of details regarding procedures in place to ensure that the allocation sequence was concealed until participants were enrolled and assigned to interventions, which rendered it impossible to evaluate whether randomisation was truly random. Another key methodological limitation in 10 (62.5%) of the trials was the relatively small samples with *N* < 60 participants.

## 4 DISCUSSION

This systematic review by the ISBD Targeting Cognition Task Force provides an updated overview of intervention trials targeting cognitive impairments in fully or partially remitted patients with mood disorders published after two previous systematic reviews in 2015 in BD and MDD, respectively.^26^, ^27^ We did not conduct a quantitative meta‐analysis of the evidence because of the discrepancies between interventions, study designs and treatment schedules. In total, 16 RCTs were identified; six involved cognitive remediation (CR), three involved direct current or repetitive magnetic stimulation (tDCS and rTMS) and seven involved pharmacological interventions, including methylene blue, DHA, modafinil, lurasidone, pramipexole and vortioxetine. The most consistent evidence for pro‐cognitive effects comes from the CR studies, of which two were formally positive, while four showed promising effects on secondary or tertiary outcomes. In contrast, the tDCS and rTMS studies showed no evidence for efficacy on cognition. Two pharmacological interventions with modafinil and lurasidone also showed cognitive benefits. Notably, the *clinical importance* of the cognitive improvement following CR interventions, lurasidone and modafinil, is unclear because it was often not accompanied by any improvement in patients’ overall functioning.

### 4.1 Methodological advancements and suggestions

Most studies (81%) were evaluated as having either moderate or high risk of bias. In addition to relatively small sample sizes, the common sources of risk of bias were the absence of information on procedures in place to ensure that the allocation sequence was masked until treatment allocation and lack of pre‐selection of one primary cognition outcome. In the previous systematic reviews of cognition trials in mood disorders published before 2015,^26^, ^27^ the risk of bias was high for 44% of the RCTs. In contrast, the risk of bias was high for only one study (6%) in the present review. This indicates an overall shift in the field towards stronger methodology. Nevertheless, some challenges remain, the most notable relating to small sample sizes. Indeed, only six (37.5%) studies had sample sizes of ≥60 patients, of which three were of CR^33^, ^35^, ^37^ and three were of pharmacological treatments with modafinil, pramipexole and vortioxetine, respectively.^43^, ^44^, ^46^ The large reported effect sizes in the CR studies for the cognitive improvements on either primary^33^ or secondary^35^, ^37^ outcomes can thus be considered relatively robust. Further, the medium to large effect size for modafinil‐induced improvement in episodic and working memory^43^ therefore also seems robust – as do the negative findings in the pramipexole and vortioxetine studies.^44^, ^46^ In contrast, the remaining 62.5% of trials may have had suboptimal statistical power and their predominantly negative findings should therefore be considered with caution.

The high frequency of small samples may reflect the early stage of the field, limited funding allocated to these mostly *investigator*‐*initiated* trials with no involvement from the pharmaceutical industry (81% of trials) and suboptimal infrastructure for recruitment (i.e., single‐site vs. multi‐site). Indeed, the largest study with *n* = 151 patients was designed and funded by Lundbeck and recruited patients from 17 psychiatric sites across five EU countries.^44^ This indicates a need for stronger multi‐site collaborations to boost sample sizes in future cognition trials. A good example is the national scientific network for mental health research, CIBERSAM, that includes 23 clinical, preclinical and translational research groups from eight communities in Spain.^50^ Indeed, this network enabled several large‐scale studies, including a functional remediation study in BD that included 239 patients.^51^ We therefore encourage national and, if possible, international collaborations, to ensure larger‐scale cognition trials with adequate power.^52^ Moreover, it would be helpful to have a clear regulatory pathway for drug approval in this indication (cognitive improvement) in the context of mood disorders. This is only in place in some countries, and not for mood disorders but rather for dementia and schizophrenia. A better roadmap for marketing authorisation would likely stimulate research from pharmaceutical companies in this field.

### 4.2 Cognitive remediation and strategies to aid transfer

The goal of CR is to improve *functional* outcome through training to remediate cognitive deficits.^53^ Consequently, CR involves both direct training of cognitive functions and compensatory strategy learning, and/or transfer to real‐world situations. In particular, transfer is essential to aid patients’ application of trained skills to tackle cognitive challenges in daily life.^53^ Nevertheless, only one CR study found improvement in patients’ psychosocial functioning and functional capacity,^37^ while two studies found improvements in self‐reported cognition and psychological function, respectively.^34^, ^35^ This lack of robust CR‐related improvement of overall functioning is noteworthy because it puts into question the *clinical impact* of the interventions.

There are several possible reasons for the limited transfer effects to community functioning. First, patients’ psychosocial impairments have multifactorial causes, with cognition being only one determinant.^53^ Second, the instruments to measure functional changes in patients tend to index more severe levels of disability, pushing functional outcomes closer to ceiling for clinically remitted patients. Third, cognitive function is measured with *performance*‐*based* neuropsychological tests, whereas functioning is often estimated based on *clinical interviews* or *self*‐*ratings*, which may be influenced by a range of factors, including depression symptoms, level of insight, personality and social support. In keeping with this, studies have generally found no or only small correlations between cognition and functioning in patients with mood disorders.^54^ Finally, as noted by Lewandowski and colleagues,^33^ it seems insufficient to merely *discuss* with patients in CR how strategies can be applied in their daily life. Indeed, computerised training games show little resemblance to daily life challenges, such that the skills acquired therefore cannot be readily applied to daily life without therapist techniques to facilitate transfer, such as role‐plays and goal setting.^55^ Thus, it is pertinent that future CR involve specific and explicit implementation of strategies to aid transfer of cognitive improvements to daily life functioning. This is in line with meta‐analytic evidence from CR trials in schizophrenia spectrum disorders that the integration of *structured psychosocial rehabilitation* with CR improves transfer of cognitive gains into real‐world settings.^56^ Future trials in mood disorders are thus warranted to investigate whether a *combination* of functional remediation or vocational training with CR can increase transfer to daily life functioning, or whether CR programmes that embed techniques to facilitate transfer within the sessions have larger effects on psychosocial function.

### 4.3 Global or selective cognition outcomes?

We previously recommended a global cognitive composite as primary outcome in cognition trials.^25^ Six studies (38%) had defined global cognition as the primary outcome^33^, ^34^, ^35^, ^39^, ^46^, ^47^ (Tables 1 and 2). This marks a clear progress from RCTs published before 2015, for which global cognition was the primary outcome in none of the trials in BD^26^ and in only three (11%) trials in MDD.^27^ The reason for the recommendation is partly that a broad cognitive composite score can detect small cumulative treatment effects across several cognitive tests. For example, a large improvement in the MCCB composite was observed in the trial by Lewandowski and colleagues despite no significant effect on *individual* MCCB tests, except visual learning.^33^ Another reason is that improvement in global (vs. specific) cognition measures is more likely to relate to improved functioning. In keeping with this, the Food and Drug Administration (FDA) encourages the use of the MCCB cognition composite as the primary outcome in cognition trials in schizophrenia partly due to its presumed correlation with the functional capacity. Nevertheless, there are situations where a specific cognitive domain or test may be preferable as the primary outcome, namely when a treatment is believed to target a specific aspect of cognition. For example, in CR, executive function is often a core component of what is being trained and seems to be the domain that is most consistently improved across CR trials.^30^ Based on this evidence as well as the direct influence of executive functions on real‐world functioning^57^ and clinical outcomes,^22^ this domain may thus be optimal as a primary or co‐primary outcome in CR trials in which training of executive functions is a core component.

### 4.4 Pre‐screening for cognitive impairment

Perhaps the most important recommendation in our prior Task Force report was to pre‐screen trial participants for *objective* cognitive performance deficits, to avoid enrolment of patients with no objective impairments and, hence, limited scope for improvement.^25^ This is particularly important for trials in mood disorders because a large proportion of these patients present with *subjective* cognitive difficulties without corresponding *objective* cognitive difficulties. However, objective cognitive pre‐screening was conducted in only five (31%) trials.^34^, ^35^, ^44^, ^46^, ^47^ We therefore reiterate the importance of pre‐screening for objective cognitive performance deficits when designing a cognitive trial in patients with mood disorders.

Importantly, emerging evidence indicates that greater impairment *within the targeted cognitive domain* is related to greater treatment benefits in that domain.^35^, ^58^, ^59^ As an update of our previous recommendation, we therefore recommend that patients are pre‐screened (i) for *broad* cognitive impairments in trials that select a *global* cognition composite as the primary outcome or, alternatively, (ii) for *specific* deficits in the domain selected as the primary outcome in studies of interventions with a purported specific cognitive target. Notably, efficacy of pro‐cognitive interventions on global cognition may be more difficult to identify if mixed samples of patients with global and selective impairments, or of patients with only selective impairments, are included. The recommendation would be to use a different – or a parallel ‐ version of the battery as a screener than the one used as the primary outcome. To screen for impairment in a particular domain, we recommend the use of several tests (rather than a single measure) that tap into this domain. This is because performance on a single test would be more prone to random variability associated with, for example, subsyndromal symptoms, anxiety or sleep difficulties.

### 4.5 Biological interventions: Preliminary targets

The identified neuromodulation studies provided mixed and preliminary evidence. The rationale for investigating potential pro‐cognitive effects of tDCS and rTMS is their assumed induction of neuroplastic changes through adjustment of the strength of synaptic transmission^60^ and evidence for working memory enhancing effects in schizophrenia.^61^ Specifically, tDCS is presumed to enhance excitatory synaptic transmission by stimulating cortical glutamate and suppressing gamma‐aminobutyric acid transmission and modulating monoamine and acetylcholine expression.^62^ However, their neurobiological mechanisms are still unclear and the evidence from the identified trials must be considered with caution.

Two^43^, ^47^ of the seven pharmacological studies showed some cognitive benefits. Acute administration of modafinil improved episodic memory and working memory,^43^ while 6 weeks of lurasidone improved global cognition.^47^ The effects of modafinil were observed with an *acute* administration in a highly controlled setting, which renders it unclear whether *longer*‐*term* modafinil treatment is safe or would induce lasting cognitive improvements. The cognitive benefits are likely to result from increased wakefulness due to stimulation of the histamine, noradrenaline, serotonin, dopamine and orexin systems.^63^ While some evidence suggests that modafinil may also have neuroprotective effects,^63^ such effects would only occur on a longer timescale. The cognitive improvement after lurasidone treatment^47^ should also be interpreted with caution because of the small sample size (*N* = 30) and lack of a double‐blind, placebo‐controlled design. Lurasidone is a full antagonist at dopamine D2 and serotonin 5‐HT2A and 5‐HT7 receptors and a partial agonist at the 5‐HT1A receptor,^64^ which are purported mechanisms of cognitive benefits for some neuroleptic drugs.^65^ While preliminary, the lurasidone‐associated cognitive improvement thus provides hypothesis‐generating evidence for cognitive benefits of prolonged modulation of serotonin and dopamine signalling.

Other promising pharmacological targets identified in previous systematic reviews of RCTs in mood disorders^26^, ^27^ are: (i) the first‐line Alzheimer's medication, galantamine, that inhibits breakdown of the enzyme acetylcholinesterase, (ii) the precursor for phosphatidylcholine synthesis, citicoline, that reduces cell‐membrane breakdown during ischaemia, hypoxia and glutamate‐mediated injury, (iii) the glucose controlling hormone, insulin, that may attenuate cerebral metabolic dysregulation, (iv) the natural herb, *Withania somnifera*, that has putative neuroprotective actions, (v) the corticosteroid receptor antagonist, mifepristone, that may counteract brain effects of hyper‐cortisolaemia, and (vi) the multifunctional glycoprotein, EPO, that has neuroprotective and neurotrophic effects. In particular, the effects of mifepristone and EPO prevailed after Bonferroni correction for multiple comparisons, rendering these particularly promising.^26^, ^27^

### 4.6 Future directions

Regarding directions for future cognition trials, a next important step will be to conduct *multimodal interventions* investigating the effects of combined treatments versus placebo/sham/TAU. This could be a combination of CR with functional or vocational training to aid transfer effects or of CR with pharmacological or other biological interventions that have shown some (even preliminary) cognitive benefits. Such multimodal interventions may, through complementary actions, have synergistic effects on neuroplasticity and cognition. Another promising strategy is the integration of strategies to improve sleep quality, such as therapy that targets sleep/social rhythms or chronotherapeutics, in combination with CR, to aid patients’ acquisition and consolidation of trained cognitive skills. Lifestyle‐based interventions (physical activity/exercise, nutrition/diet) may also – either alone or in combination^66^ – be implemented in a multimodal intervention to facilitate neuroplasticity and cognitive functions.^67^, ^68^ Indeed, the heightened risk of cardiovascular disease, diabetes and dementia in mood disorders^69^ supports the implementation of such lifestyle interventions in such multimodal interventions targeting cognition. In keeping with this, the inclusion of physical exercise as an integral part of multimodal pro‐cognitive interventions^70^ shows promising results in schizophrenia^71^ and symptomatic MDD.^72^

Finally, a recommended next step is the implementation of *neuroimaging* to investigate whether candidate pro‐cognitive treatments target the aberrant neurocircuitry activity and structural abnormalities that underlie cognitive impairments.^73^, ^74^ This will likely reveal neurocircuitry‐based biomarkers that may be useful tools in treatment development strategies to screen and select among novel candidate treatments in small clinical phase 2 trials prior to commencing large‐scale costly phase 3 trials.

### 4.7 Limitations

The lack of a quantitative meta‐analysis of the effect sizes of treatment‐related cognitive improvements was a limitation. However, this was due to the discrepancies between types of interventions, study designs and treatment schedules in the trials. Rather, our aim was to update and evaluate the quality of the evidence from RCTs and, based on this, provide updated methodological recommendations. The restriction of our search to RCTs published between 2015 and 2021 prevented a more comprehensive overview of the field. Nevertheless, an extension of the inclusion dates would have led to duplication of previous findings rather than a focused up‐to‐date review of the most recent evidence and current methodological challenges. The focus on remitted patients may be considered a limitation since head‐to‐head studies in non‐remitted patients could also reveal key insights into potential pro‐cognitive treatments. Specifically, if the comparison of two active treatments in acutely depressed MDD patients reveals equal antidepressant effects but greater cognitive benefits of one treatment, then this would provide promising evidence for pro‐cognitive efficacy of this intervention. Such head‐to‐head trials with cognition as primary endpoint might be informative even if conducted with non‐remitted patients, since pseudospecificity would in this way be controlled by the active comparator design,^75^ as exemplified by vortioxetine trials in symptomatic MDD.^76^ Nevertheless, such designs are not straight forward as it is not clear whether superiority or non‐inferiority designs, influencing statistical power and sample sizes, should be preferred in relation to the antidepressant and pro‐cognitive effects. Limiting the review to remitted patients can thus be considered a strength, as this addresses treatment‐related improvement in the persistent trait‐related cognitive deficits with a long‐lasting negative impact on patients’ functioning.

### 4.8 Conclusions and recommendations

In conclusion, this updated systematic review of RCTs published between 2015 and 2021 identified 16 RCTs in partially or fully remitted patients with BD or UD. Six studies involved CR, three tDCS or rTMS and seven pharmacological interventions, including methylene blue, DHA, modafinil lurasidone, pramipexole and vortioxetine. Most consistent cognitive improvements were observed with CR, with two trials being formally positive and four showing preliminary effects. In contrast, the tDCS and rTMS studies showed no cognitive benefits. Among pharmacological interventions, modafinil and lurasidone showed some cognitive benefits. Most studies had moderate risk of bias due to several common methodological challenges. As a supplement to our previous consensus‐based recommendations,^25^ we suggest that future cognition trials include: (i) increased sample sizes in trials through national and international collaborations when possible, (ii) pre‐selection of one cognition outcome as primary, (iii) pre‐screening for cognitive impairments within the targeted domain(s), (iv) strategies to aid transfer of cognitive gains to patients’ daily lives, (v) adequate reporting of procedures for masking the allocation sequence, (vi) multimodal interventions and (vii) neuroimaging or other biomarkers to assess neurocircuitry target engagement. See the complete updated Task Force recommendations including our previous and newly added recommendations in Table 3. These include also recommendations regarding how to handle concomitant medication and specific classes of agents, criteria to select trial participants, how to define a ‘clinically relevant’ cognitive improvement, when to conduct pre‐ and post‐assessments and how to handle statistical issues around missing data. Following these recommendations will likely improve the chances of identifying effective pro‐cognitive treatments in RCTs and, thereby, accelerate the rate at which they can be integrated in the clinical management of mood disorders.

**TABLE 3:** Updated methodological recommendations for pro‐cognitive intervention trials in mood disorders by the International Society for Bipolar Disorders Targeting Cognition Task Force

Quick guide
How can we enrich trials with cognitively impaired patients? Pre‐screen participants for *objective* cognitive impairments with a brief cognition screening battery Pre‐screen for either (i) *broad* cognitive impairments in trials for which a global cognitive composite is the primary outcome or (ii) *specific* deficits in a particular cognitive domain in studies of interventions with a purported specific cognitive target Use a *different* cognitive test battery to (or a parallel version of) the cognitive test battery implemented as the primary outcome To screen for impairment in a particular domain, use of several tests (rather than a single test) that tap into this domain
What is a feasible threshold for cognitive impairment? ≥0.5 SD below the normative mean for a cognitive composite based on an objective cognition screener or ≥1 SD below the mean on ≥2 single cognitive tests If logistically feasible, cognitive impairment may be established with reference to general IQ
Which criteria should be used to select trial participants? Generally, include partially or fully remitted patients in trials where cognition is primary outcome to minimise ‘pseudospecificity’ issues Exclude patients with a history of moderate or severe brain injury, neurological disease, current uncontrolled thyroid condition, unstable medical illness, current or recent alcohol and substance use disorders, intellectual disability, or ECT within the past 6 months Allow concomitant medications. These should be carefully recorded and, if possible, kept stable In possible, disallow certain medications (e.g., high‐dose antipsychotics and anticholinergic medications) Taper benzodiazepines to a maximum dose equivalent to 22.5 mg oxazepam/7.5 mg diazepam per day and restrict use of benzodiazepine and other hypnotics six hours prior to cognitive testing Keep serum lithium levels within the therapeutic range
How should efficacy on cognition be assessed? Pre‐select *one cognition measure* as the primary outcome In general, the primary outcome should be a *broad* cognitive composite score spanning attention, verbal memory, and executive functions. Alternatively, in trials targeting a specific cognitive domain, this would ideally be a composite score based on several tests (rather than one test) tapping into this domain Use tests that are broadly equivalent to those included in the ISBD‐BANC^49^ Select key cognitive tests of interest and a functional measure as secondary outcomes
What is a ‘clinically relevant’ cognitive improvement? Since learning effects are almost impossible to eliminate, a ‘clinically relevant’ effect on cognition should be estimated with reference to the cognitive change in the control group Given the issue with learning effects (which reduce the difference between the active and control groups), small to medium effect sizes for treatment effects may be considered clinically meaningful
How should functional implications be evaluated? The FAST, UPSA‐B and VRFCAT are among the best measures to date for tracking changes in functional capacity associated with cognitive improvement in bipolar disorder
How should we support transfer of cognitive gains to patients’ daily lives? Combine pro‐cognitive interventions (CR or biological treatments) with functional remediation or vocational training Implement techniques to facilitate transfer within the CR programmes, such as role‐play and goal setting
When should pre‐ and post‐assessments be conducted? The optimal duration of a particular trial depends on the presumed onset of efficacy for the particular intervention based on its putative mechanisms In general, administer biological interventions for 6–12 weeks and psychological interventions for 10–21 weeks with pre‐ and post‐treatment assessments of cognition at baseline and immediately after treatment completion. If feasible, perform follow‐up assessments after 3–6 months
How should ‘pseudospecificity’ be addressed? Adjust the statistical analysis of cognitive change for symptom fluctuation and conduct path analysis
What are the methodological recommendations for specific classes of agents? Monotherapy should only be used if the candidate treatment has mood stabilising effects for ethical reasons and to ensure generalisability. Use an active comparator drug with mood stabilising effects Cognition trials investigating anti‐psychotic, pro‐dopaminergic or antidepressant drugs with efficacy on depressive symptoms should *ideally* include euthymic patients to rule out pseudospecificity. Alternatively, they can include depressed patients in a head‐to‐head adjunctive superiority design with a comparator without pro‐cognitive effects Trials investigating anti‐inflammatory or neuroprotective drugs with limited effects on mood would benefit from expanding the inclusion criteria to partial remission in the interest of recruitment feasibility and generalisability. Use an adjunctive study design with a placebo control
How can we ensure adequate sample sizes and, hence, optimised statistical power? Increase sample sizes through national and international collaborations when possible Improving clarity on the regulatory pathway for drug approval for cognitive improvement in the context of mood disorders may also attract greater interest – and financial support – from the pharmaceutical industry
How should statistical issues around missing data be handled? Intention‐to‐treat analyses should be implemented to prevent bias caused by dropout Feasible ways to handle missing data with repeated assessments after treatment start are multiple imputation or mixed models
Multimodal interventions particularly promising: why and how? Multimodal treatments may through synergistic effects produce stronger, longer‐lasting improvements The primary goal would be to investigate the effects of multimodal treatment versus placebo/sham/TAU Examples are a combination of CR with: (i) functional/vocational training, (ii) biological interventions that have (even preliminary) benefits, (iii) strategies to improve sleep quality, or (iv) lifestyle‐based interventions
Neuroimaging assessments in treatment trials: why and how? If possible, implement neuroimaging assessments (e.g., before and after interventions) to investigate whether candidate pro‐cognitive treatments target the aberrant neurocircuitry activity and structural abnormalities that underlie cognitive impairments This will likely reveal neurocircuitry‐based biomarkers that may be useful tools in treatment development strategies to improve the success rates of treatment trials

## CONFLICT OF INTEREST

KWM has received consultancy fees from Janssen and Lundbeck in the past 3 years. RP uses computer software at no cost for research – provided by SBT‐pro and has received support for travel to educational meetings from Servier and Lundbeck. AS has received advisory or speaking fees from AbbVie, Janssen, Lundbeck, Otsuka and Sunovion in the past 3 years. KD uses computer software at no cost for research provided by SBT‐pro. LVK has received consultancy fees from Lundbeck and Teva in the past 3 years. EV has received grants and served as consultant, advisor or CME speaker for the following entities: AB‐Biotics, Abbott, Allergan, Angelini, Dainippon Sumitomo Pharma, Ferrer, Gedeon Richter, Janssen, Lundbeck, Otsuka, Sage, Sanofi‐Aventis, Sunovion, Takeda, all them unrelated to the present work. VBM has been a consultant, advisor or Continuing Medical Education (CME) speaker over the last 3 years for the following companies: Angelini, Lundbeck, Nutrición Médica and Otsuka. AY has conducted paid lectures and advisory boards for Allergan, AstraZeneca, Bionomics, BrainCells Inc., Bristol‐Myers Squibb, Eli Lilly, GlaxoSmithKline, Janssen, Lundbeck, Novartis, Otsuka Pharmaceutical Co., Pharmaceutica, Pfizer, Roche, Sanofi‐Aventis, Servier Laboratories, Sunovion and Wyeth. He was lead Investigator for the EMBOLDEN Study (AstraZeneca), BCI Neuroplasticity Study and Aripiprazole Mania Study, and has been involved in investigator‐initiated studies for AstraZeneca, Eli Lilly and Wyeth. PS reports grants and non‐financial support from Corcept Therapeutics, non‐financial support from Janssen Research and Development LLC, grant funding from Lundbeck and personal fees from Frontiers in Psychiatry and Allergan outside the submitted work. IJT has received has served as consultant for Lundbeck Canada, Sumitomo Dainippon and Community Living British Columbia. LNY has been on speaker/advisory boards for, or has received research grants from Alkermes, Allergan, AstraZeneca, Bristol Myers Squibb, CANMAT, CIHR, Dainippon Sumitomo Pharma, GSK, Janssen, Lilly, Lundbeck, Merck, Otsuka, Pfizer, Sanofi, Sunovion and Teva. RM has received personal fees from Lundbeck, Janssen, Purdue, Pfizer, Otsuka, Allergan, Takeda, Neurocrine, Sunovion, Minerva, Intra‐Cellular, Abbvie and Eisai and is a shareholder in the 420 Company and CEO of Champignon. AD, AC, BL, IS, MBJ, CRB, CMB, KEL, PG, CLJ, AMA, SEP, TS and TVR report no conflict of interest.

## Supporting information

Supplementary Material
