# Supplementary material for: Randomised controlled cognition trials in remitted patients with mood disorders published between 2015 and 2021: A systematic review by the International Society for Bipolar Disorders Targeting Cognition Task Force
Source: Bipolar Disord. 2022 Feb 24;24(4):354–74. doi: 10.1111/bdi.13193 (PMC9541874; doi:10.1111/bdi.13193)
Supplement: Supplementary file 1 — Supplementary Material [file BDI-24-354-s001.docx]

**Supplementary material**

**Search profile on PubMed February 2021**

|  | Group: BD+UD | Focus: cognition | Focus:  Intervention | Focus:  RCT | Focus:  Publication year 2015-now |  |  |
| --- | --- | --- | --- | --- | --- | --- | --- |
| MeSH | #1 | #2 | #3 | #4 | #5 | #6 | 567 hits |
| Ti/Ab | #7 | #8 | #9 | #10 | #11 | #12 | 2925 hits |
| NOT MEDLINE [SB] |  |  |  |  |  | #13 | 901 hits |

***Search lines PubMed***

#1: Search: "Mood Disorders"[Mesh] OR "Bipolar Disorder"[Mesh] OR "Depression"[Mesh]

#2: Search: "Cognitive Dysfunction"[Mesh] OR "Cognition"[Mesh] OR "Executive Function"[Mesh] OR "Attention"[Mesh] OR "Memory"[Mesh] OR "Cognition Disorders" [Mesh]

#3: Search: "Therapeutics"[Mesh] OR "therapy" [Subheading]

#4: Search: "Randomized Controlled Trial" [Publication Type] OR "Clinical Trial" [publication type]

#5: Search: "2015/01/01"[Date - Publication] : "3000"[Date - Publication]

#6: Search: (("Mood Disorders"[MeSH Terms] OR "Bipolar Disorder"[MeSH Terms] OR "Depression"[MeSH Terms]) AND ("Cognitive Dysfunction"[MeSH Terms] OR "Cognition"[MeSH Terms] OR "Executive Function"[MeSH Terms] OR "Attention"[MeSH Terms] OR "Memory"[MeSH Terms] OR "Cognition Disorders"[MeSH Terms]) AND ("Therapeutics"[MeSH Terms] OR "therapy"[MeSH Subheading]) AND ("Randomized Controlled Trial"[Publication Type] OR "Clinical Trial"[Publication Type])) AND ("2015/01/01"[Date - Publication] : "3000"[Date - Publication])

#7: Search: bipolar[Title/Abstract] OR unipolar[Title/Abstract] OR depressi*[Title/Abstract] OR affective disorder*[Title/Abstract] OR mood disorder*[Title/Abstract] OR maniodepressi*[Title/Abstract]

#8: Search: cogniti*[Title/Abstract] OR procogniti*[Title/Abstract]

#9: Search: therapy[Title/Abstract] OR psychoeducation[Title/Abstract] OR

treat[Title/Abstract] OR treating[Title/Abstract] OR treatment[Title/Abstract] OR treated[Title/Abstract] OR pharmacotherapy[Title/Abstract] OR

psychotherapy[Title/Abstract] OR intervention[Title/Abstract] OR

psychoanalysis[Title/Abstract]

#10: Search: randomised placebo control*[Title/Abstract] OR randomized placebo control*[Title/Abstract] OR randomised control*[Title/Abstract] OR randomized control*[Title/Abstract]

#11: Search: ("2015/01/01"[Date - Publication] : "3000"[Date - Publication])

#12: Search: ("bipolar"[Title/Abstract] OR "unipolar"[Title/Abstract] OR "depressi*"[Title/Abstract] OR "affective disorder*"[Title/Abstract] OR "mood disorder*"[Title/Abstract] OR "maniodepressi*"[Title/Abstract]) AND ("cogniti*"[Title/Abstract] OR "procogniti*"[Title/Abstract]) AND ("therapy"[Title/Abstract] OR "psychoeducation"[Title/Abstract] OR "treat"[Title/Abstract] OR "treating"[Title/Abstract] OR "treatment"[Title/Abstract] OR "treated"[Title/Abstract] OR "pharmacotherapy"[Title/Abstract] OR "psychotherapy"[Title/Abstract] OR "intervention"[Title/Abstract] OR "psychoanalysis"[Title/Abstract]) AND ("randomised placebo control*"[Title/Abstract] OR "randomized placebo control*"[Title/Abstract] OR "randomised control*"[Title/Abstract] OR "randomized control*"[Title/Abstract]) AND 2015/01/01:3000/12/31[Date - Publication]

#13: Search: (("bipolar"[Title/Abstract] OR "unipolar"[Title/Abstract] OR "depressi*"[Title/Abstract] OR "affective disorder*"[Title/Abstract] OR "mood disorder*"[Title/Abstract] OR "maniodepressi*"[Title/Abstract]) AND ("cogniti*"[Title/Abstract] OR "procogniti*"[Title/Abstract]) AND ("therapy"[Title/Abstract] OR "psychoeducation"[Title/Abstract] OR "treat"[Title/Abstract] OR "treating"[Title/Abstract] OR "treatment"[Title/Abstract] OR "treated"[Title/Abstract] OR "pharmacotherapy"[Title/Abstract] OR "psychotherapy"[Title/Abstract] OR "intervention"[Title/Abstract] OR "psychoanalysis"[Title/Abstract]) AND ("randomised placebo control*"[Title/Abstract] OR "randomized placebo control*"[Title/Abstract] OR "randomised control*"[Title/Abstract] OR "randomized control*"[Title/Abstract]) AND 2015/01/01:3000/12/31[Date - Publication]) NOT "MEDLINE"[Filter]

**Search profile PsycInfo February 2021**

|  | Group: BD+UD | Focus: cognition | Focus:  treatment | Focus:  RCT | Focus:  Publication year 2015-now |  |  |
| --- | --- | --- | --- | --- | --- | --- | --- |
| Ti/Ab | #1 | #2 | #3 | #4 | #5 | #6 | 1593 hits |

#1: TI (Bipolar OR Unipolar OR depressi* OR affective disorder* OR Mood disorder* OR Maniodepressi*) OR AB (Bipolar OR Unipolar OR Depressi* OR Affective Disorder* OR Mood Disorder* OR Maniodepressi*)

#2: TI (Cogniti* OR Procogniti*) OR AB (Cogniti* OR TI Procogniti*)

#3: TI (therapy or psychoeducation or treat or treating or treatment or treated or pharmacotherapy or psychotherapy or intervention or psychoanalysis) OR AB (therapy or psychoeducation or treat or treating or treatment or treated or pharmacotherapy or psychotherapy or intervention or psychoanalysis)

#4: TI randomi?ed W3 control* OR AB randomi?ed W3 control*

#5: Limiters - Publication Year: 2015-

#6: (TI (Bipolar OR Unipolar OR depressi* OR affective disorder* OR Mood disorder* OR Maniodepressi*) OR AB (Bipolar OR Unipolar OR Depressi* OR Affective Disorder* OR Mood Disorder* OR Maniodepressi*)) AND (TI (Cogniti* OR Procogniti*) OR AB (Cogniti* OR TI Procogniti*)) AND (TI (therapy or psychoeducation or treat or treating or treatment or treated or pharmacotherapy or psychotherapy or intervention or psychoanalysis) OR AB (therapy or psychoeducation or treat or treating or treatment or treated or pharmacotherapy or psychotherapy or intervention or psychoanalysis)) AND (TI randomi?ed W3 control* OR AB randomi?ed W3 control*) AND (Limiters - Publication Year: 2015-)

## Search Profile EMBASE & Cochrane library February 2021

|  | Group: BD+UD | Focus: cognition | Focus:  intervention | Focus:  RCT | Focus:  Publication year 2015-now |  |  |
| --- | --- | --- | --- | --- | --- | --- | --- |
| Terms | #1 | #2 | #3 | #4 | #5 | #6 | 49 hits |
| Title | #7 | #8 | #9 | #10 | #11 | #12 | 62 hits |

***Search lines EMbase & Cochrane library***

#1: exp depression/ or exp mania/

#2: cognition/ or attention/ or memory/ or executive function/

#3: exp psychiatric treatment/

#4: exp randomized controlled trial/

#5: yr="2015 -Current"

#6: limit 5 to yr="2015 -Current" (where 5 = 1 and 2 and 3 and 4)

#7: (bipolar or unipolar or depressi* or affective disorder* or mood disorder*).m_titl.

#8: (cogniti* or procogniti*).m_titl.

#9: (therapy or psychoeducation or treat or treating or treatment or treated or pharmacotherapy or psychotherapy or intervention or psychoanalysis).m_titl.

#10: ("randomised placebo control*" or "randomized placebo control*" or "randomised control*" or "randomized control*").m_titl.

#11: yr="2015 -Current"

#12: Limit 11 to yr="2015 -Current" (where 11 = 7 and 8 and 9 and 10)
